# Disagreement between Human Papillomavirus Assays: An Unexpected Challenge for the Choice of an Assay in Primary Cervical Screening

**DOI:** 10.1371/journal.pone.0086835

**Published:** 2014-01-20

**Authors:** Matejka Rebolj, Sarah Preisler, Ditte Møller Ejegod, Carsten Rygaard, Elsebeth Lynge, Jesper Bonde

**Affiliations:** 1 Department of Public Health, University of Copenhagen, Copenhagen, Denmark; 2 Department of Pathology, Copenhagen University Hospital Hvidovre, Hvidovre, Denmark; 3 Clinical Research Centre, Copenhagen University Hospital Hvidovre, Hvidovre, Denmark; Centers for Disease Control and Prevention, United States of America

## Abstract

We aimed to determine the disagreement in primary cervical screening between four human papillomavirus assays: Hybrid Capture 2, cobas, CLART, and APTIMA. Material from 5,064 SurePath samples of women participating in routine cervical screening in Copenhagen, Denmark, was tested with the four assays. Positive agreement between the assays was measured as the conditional probability that the results of all compared assays were positive given that at least one assay returned a positive result. Of all 5,064 samples, 1,679 (33.2%) tested positive on at least one of the assays. Among these, 41% tested positive on all four. Agreement was lower in women aged ≥30 years (30%, vs. 49% at <30 years), in primary screening samples (29%, vs. 38% in follow-up samples), and in women with concurrent normal cytology (22%, vs. 68% with abnormal cytology). Among primary screening samples from women aged 30–65 years (n = 2,881), 23% tested positive on at least one assay, and 42 to 58% of these showed positive agreement on any compared pair of the assays. While 4% of primary screening samples showed abnormal cytology, 6 to 10% were discordant on any pair of assays. A literature review corroborated our findings of considerable disagreement between human papillomavirus assays. This suggested that the extent of disagreement in primary screening is neither population- nor storage media-specific, leaving assay design differences as the most probable cause. The substantially different selection of women testing positive on the various human papillomavirus assays represents an unexpected challenge for the choice of an assay in primary cervical screening, and for follow up of in particular HPV positive/cytology normal women.

## Introduction

Screening for human papillomavirus has better sensitivity for high-grade cervical intraepithelial neoplasia and provides protection against cervical cancer for a longer time than cytology screening [1]–[3]. This was demonstrated in studies using predominantly the Digene Hybrid Capture 2® HPV Test (Qiagen, Gaithersburg, MD), and GP5+/6+ polymerase chain reaction assays. However, several human papillomavirus assays have since become commercially available, and well-documented comparative studies are needed for laboratories to select the most appropriate assay for primary screening.

The proposed management strategies for women testing positive for human papillomavirus have so far been based on evidence from a small number of assays. Until now, the newly commercially available assays have most often been compared against Hybrid Capture 2 in women with recent cytological abnormalities [4]–[18]. In routine screening, however, women with cytological abnormalities constitute a selected population, whereas a majority of positive human papillomavirus samples are from women without cytological abnormalities. Hence, studies of women with cytological abnormalities cannot capture the diversity of outcomes of human papilomavirus testing in primary screening. Furthermore, the few primary cervical screening studies comparing various assays used relatively crude outcome measures, e.g. kappa coefficients, and suggested good overall agreement [15], [19]–[24]. However, to determine whether the management strategies for women with positive tests are applicable to other assays, more detailed analyses of outcomes from the various assays are needed. A first step is simply to know whether the same women test positive on different human papillomavirus assays.

The Horizon study was a population based split sample study comparing Hybrid Capture 2, cobas® HPV Test (Roche Diagnostics, Pleasanton, CA), CLART® HPV2 Assay (Genomica, Madrid, Spain), and APTIMA® HPV Test (Hologic/Gen-Probe, San Diego, CA; Table 1). It was undertaken on routine samples from a well screened population. The aim of this analysis was to determine the frequency of disagreement between the four human papillomavirus assays, particularly in primary screening.

**Table 1:** Human Papillomavirus assays compared in the Horizon study.

Human papillomavirus assay	Targeted human papillomavirusgenotypes	Detection technology	DNA/RNA, and the targeted HPV gene	Controls	Certificates
**APTIMA**	13 HR + 1 LR	MMLV reverse transcriptaseand T7 RNA polymerasemediated amplification	RNA, *E6/E7*	Spiked control for sample processvalidity	FDA, IVD, CE
**CLART**	13 HR + 22 LR	Polymerase chain reaction/microarray	DNA, *L1*	Sample by sample hCFTR gene forsample sufficiency	IVD, CE
**Cobas**	13 HR + 1 LR	Real-time polymerase chain reaction	DNA, *L1*	Sample by sampleβ-globin for samplesufficiency	FDA, IVD, CE
**Hybrid Capture 2**	13 HR	Sandwich capture molecular	DNA, *whole genome*	Batch control for process	FDA, IVD, CE

Abbreviations: CE = Conformité Européenne mark; FDA = US Food and Drug Administration; HR = high risk genotypes using the International Agency for Research on Cancer’s classification, including the “probably carcinogenic” genotype 68 [26]; IVD = in vitro diagnostic medical device; LR = low risk genotypes using the International Agency for Research on Cancer’s classification, including the “possibly carcinogenic” genotype 66 [26].

## Materials and Methods

### Setting

The Department of Pathology at Hvidovre University Hospital in Copenhagen, accredited by the Joint Commission International, handles all cervical cytology from central Copenhagen. Copenhagen has been covered by an organized cervical screening program since the 1960s. Currently, women aged 23 to 49 years are invited for screening every three years, and women aged 50 to 65 years are invited every five years; in recent years, 76% of women had cytology in the recommended interval [25].

### Sample Collection

Horizon was a quality development study nested into routine laboratory practice, and utilized only residual material that would have otherwise been discarded. According to Danish regulations of biomedical research, quality development studies do not require ethical approval.

Upon arrival at the laboratory, consecutive samples were collected in racks of 48. They were collected from 10 June to 25 August 2011, equally from Monday to Friday. Approximately 2 ml of residual material were collected after completion of routine SurePath liquid based cytology and Hybrid Capture 2 triage of women aged ≥30 years with atypical squamous cells of undetermined significance. Samples were collected from the first four racks or fewer processed on the collection days. This method mimicked a collection of unselected consecutive samples, assuming that the time of sample arrival in the laboratory was not associated with its characteristics. Samples were diluted with 2 ml of SurePath to obtain enough volume for all four assays. Based on capacity and processing considerations, the target number of samples was set to 5,000.

From the 12,138 routine samples processed during the collection period, 6,258 (52%) were selected for Horizon. For 1,194 (19%) samples, complete human papillomavirus testing could not be undertaken: 1,165 samples were tested only with Hybrid Capture 2 owing to lack of residual material for the other three assays, whereas 29 samples could not be systematically tested on all four assays owing to human error. Consequently, 5,064 (81%) samples with cytology and complete results on the four human papillomavirus assays and cytology were included in the analysis (Table 2). A single sample was available from 5,005 (99%) women, whereas 59 samples (1%) were from the remaining 29 women.

**Table 2:** Samples collected for the Horizon study (n = 6,258), by age, screening history, and outcomes of cytology and Human Papillomavirus testing.

	Samples with complete results on all four humanpapillomavirus assays (%)^a^			Samples with only Hybrid Capture 2 results and cytology (%)^b^	P^c^
	Total	Primary	Follow-up		
	5,064 (100%)	4,407 (100%)	657 (100%)	1,194 (100%)^d^	
**Age (years)**					0.10
≤22	162 (3.2%)	135 (3.1%)	27 (4.1%)	42 (3.5%)	
23 to 29	1,534 (30.3%)	1,286 (29.2%)	248 (37.7%)	372 (31.2%)	
30 to 39	1,525 (30.1%)	1,299 (29.5%)	226 (34.4%)	360 (30.2%)	
40 to 49	991 (19.6%)	903 (20.5%)	88 (13.4%)	202 (16.9%)	
50 to 59	506 (10.0%)	464 (10.5%)	42 (6.4%)	119 (10.0%)	
60 to 65	234 (4.6%)	215 (4.9%)	19 (2.9%)	76 (6.4%)	
≥66	112 (2.2%)	105 (2.4%)	7 (1.1%)	22 (1.8%)	
Average (range)	37.3 (16 to 89)	37.8 (16 to 89)	34.1 (18 to 73)	37.2 (15 to 91)	
Median (interquartile range)	34 (27 to 45)	35 (28 to 46)	31 (26 to 39)	34 (27 to 45)	
**Screening history**					0.01
Primary	4,407 (87.0%)	NR	NR	1,072 (89.8%)	
Follow-up	657 (13.0%)	NR	NR	122 (10.2%)	
**Cytology outcome**					0.54
Normal cytology	4,667 (92.2%)	4,145 (94.1%)	522 (79.5%)	1,108 (92.8%)	
Atypical squamous cells of undetermined significance	123 (2.4%)	85 (1.9%)	38 (5.8%)	24 (2.0%)	
Low grade squamous intraepithelilal lesions	142 (2.8%)	87 (2.0%)	55 (8.4%)	30 (2.5%)	
High grade squamous intraepithelial lesions or worse^e^	106 (2.1%)	69 (1.6%)	37 (5.6%)	22 (1.8%)	
Inadequate	26 (0.5%)	21 (0.5%)	5 (0.8%)	10 (0.8%)	
**Positive human papillomavirus outcomes** f					
Cobas	1,356 (26.8%)	1,077 (24.4%)	279 (42.5%)	NT	
CLART	1,273 (25.1%)	1,023 (23.2%)	250 (38.1%)	NT	
Hybrid Capture 2	1,035 (20.4%)	822 (18.7%)	213 (32.4%)	255 (21.8%)	0.31
APTIMA	846 (16.7%)	674 (15.3%)	172 (26.2%)	NT	

Abbreviations: NR = not relevant; NT = not tested.

^a^ Included samples.

^b^ Excluded samples.

^c^ Differences between included samples (total) and excluded samples, tested with χ^2^.

^d^ Information on age was missing for one sample. Hybrid Capture 2 testing was not undertaken on 23 samples.

^e^ Including atypical squamous cells cannot exclude high grade squamous intraepithelial lessions, atypical glandular cells, adenocarcinoma in situ, and carcinoma.

^f^ Numbers (%) of invalid samples, out of 5,064: cobas: 3 (<0.1%), CLART 12 (0.2%), Hybrid Capture 2 0, APTIMA 1 (<0.1%).

### Cytology

Routine cytological evaluation of SurePath samples was undertaken first by FocalPoint Slide Profiler (BD, Burlington, NC). Blinded to the outcomes of human papillomavirus testing in Horizon, samples were thereafter evaluated by cytoscreeners using FocalPoint GS Imaging System (BD), and abnormal findings were adjudicated by pathologists. Cytology was reported using the Bethesda 2001 system.

### Hybrid Capture 2 Human Papillomavirus DNA Testing

On the post-quot material from the cytology procedure, DNA was either denatured prior to testing by pre-treating manually according to the manufacturer’s CE-IVD protocol, or DNA was isolated and purified using the DSP AXpH DNA kit on QIASymphony SP (Qiagen, Hilden, Germany). As part of the cytology processing, post-quot material was diluted approximately 1∶1 in SurePath. Testing was undertaken on automated Rapid Capture® System (Qiagen, Gaithersburg, MD, USA). A minority of samples used for routine Hybrid Capture 2 triage of women aged ≥30 years with atypical squamous cells of undetermined significance were denatured and tested manually.

### Cobas Human Papillomavirus DNA Testing

1 ml of the diluted material was aliquoted into a 13 ml round bottom test tube (Sarstedt, cat. no NC9018280), stored at 2 to 8°C until testing. No pre-treatment of SurePath samples was required. Extraction of DNA was undertaken on cobas x480, and amplification and detection of high risk human papillomavirus DNA on cobas z480 analyzer. Fluorescent TaqMan® probes were used for detection of the amplicons during polymerase chain reaction cycles. Amplification and detection of the 330 bp β-globin was used as an internal control of the testing processes.

### CLART Human Papillomavirus DNA Testing

1 ml of the diluted SurePath sample was spun down (five minutes, 14,000 revolutions per minute), with supernatant removed and cell pellet re-suspended in a mix of 180 µl phosphate buffered saline (10x conc. pH 7.4, Pharmacy product) and 20 µl Proteinase K (recombinant, PCR Grade, Roche Diagnostics, Rotkreuz, Switzerland). Samples were then vortexed and incubated for one hour at 56°C and one hour at 90°C. Human papillomavirus DNA was purified using MagNa Pure LC 96 and MagNA Pure LC 32 instruments (Roche Diagnostics) with MagNA Pure LC Total Nucleic Acid Isolation Kit (Roche Diagnostics). Polymerase chain reaction amplification was performed using CLART® HPV2 Amplification kit (Genomica). 5 µl of purified DNA were used for the polymerase chain reaction amplification. Prior to visualisation, the polymerase chain reaction products were denatured at 95°C for 10 minutes. Visualisation was performed using 10 µl of the denatured polymerase chain reaction products on the CLART microarray. Hybridisation between the amplicons and their specific probes on the microarray resulted in formation of an insoluble precipitate of peroxidase when adding a Streptavidin conjugate that binds to the biotin labelled polymerase chain reaction products. The precipitate was analyzed automatically on the Clinical Array Reader (Genomica).

### APTIMA Human Papillomavirus mRNA Testing

1 ml of the diluted sample was aliquoted into an APTIMA Specimen Transfer Tube containing 2.9 ml of buffered solution (Hologic/Gen-Probe). Samples were treated with proteinase K prior to testing, using the Pace 2 Fast Expression Kit containing 1 ml diluent and lyophilized reagent (all from Hologic/Gen-Probe). 100 µl of the reconstituted proteinase K was added to each Specimen Transfer Tube and incubated at 65°C for two hours. The treated specimen tube was stored at 2 to 8°C until testing. Testing was performed on the PANTHER platform.

### Processing of Samples and Assay Instrumentation

The study protocol, sample storage, and assay testing protocols were agreed upon with all manufacturers prior to the study. All instrumentation and software were used as supplied and maintained by the manufacturers.

### Screening History

As described above, all women were previously screened with liquid-based cytology, and those with atypical squamous cells of undetermined significance at age 30 years or above were triaged using the Hybrid Capture 2 assay. The screening history of women from 1 January 2000 onwards was retrieved from the Danish Pathology Data Bank. Following Danish recommendations for follow-up of cervical abnormalities, Horizon samples with an earlier diagnosis of cervical cancer, a diagnosis of cervical intraepithelial neoplasia in up to three years earlier, with atypical squamous cells of undetermined significance in the previous 15 months, with more severe cytological abnormalities or a positive human papillomavirus test in the past 12 months were considered follow-up samples. Samples with no recent abnormality were considered primary samples; reflecting routine practice, these included screening samples and a small proportion of samples taken by indication.

### Statistical Analysis

A positive human papillomavirus test was defined according to the manufacturers’ recommendations (Hybrid Capture 2: relative light unit per cut off value ≥1; cobas channels 16, 18, and other high risk genotypes: critical threshold values ≤40.5, ≤40.0, and ≤40.0, respectively; APTIMA: signal to cut off value ≥0.5). CLART was considered positive if at least one of the 13 human papillomavirus genotypes classified as high risk by the International Agency for Research on Cancer, including genotype 68, was detected [26]. Kappa coefficients were calculated as a standard measure of agreement for each pair of assays; their 95% confidence intervals were calculated by analysing 1,000 bootstrap replications (IBM® SPSS® Statistics, Version 20). The frequencies of positive concordant (positive on assay A/positive on assay B), and of discordant (positive/negative, negative/positive) samples were calculated separately. The sum of the proportions of discordant samples equalled [100% - proportion of overall agreement]. Positive agreement was calculated as the conditional probability that all compared assays were positive (concordant positive samples) given that at least one assay returned a positive result (concordant positive+any discordant samples), and was reported as a proportion. Its 95% confidence interval was calculated assuming binomial distribution of the studied events.

## Results

Among the 5,064 samples included in the analysis, 4,790 (94.6%) were from women targeted by the Danish cervical screening program, aged 23 to 65 years (Table 2). Cytology was abnormal in 371 (7.3%) of the 5,064 samples, cobas was positive in 1,356 (26.8%), CLART in 1,273 (25.1%), Hybrid Capture 2 in 1,035 (20.4%), and APTIMA in 846 (16.7%) samples. These proportions were higher for follow-up than for primary samples for all four assays.

**Figure 1:**
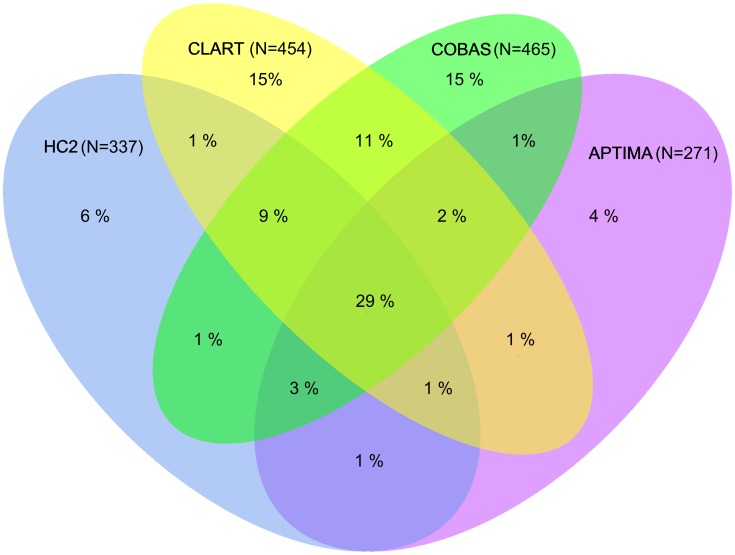
Primary samples, 30–65 years: Disagreement between Hybrid Capture 2, cobas, CLART, and APTIMA HPV assays. Proportions were calculated from all samples that tested positive on at least one assay.

Overall, 1,679 (33.2%) out of 5,064 samples were positive on at least one of the four human papillomavirus assays (Table 3). Of these 1,679 samples, 681 (41%) were positive on all four, 260 (15%) on three, 268 (16%) on two, and 470 (28%) on a single human papillomavirus assay. Positive agreement between the assays was lower for women aged 30 to 65 compared to women aged 23 to 29 years. Among women aged 30 to 65 years, positive agreement was higher for follow-up than for primary samples; disagreement among primary samples in this age group is presented in more detail on Figure 1. Among the latter samples, positive agreement was substantially higher in women with atypical squamous cells of undetermined significance or worse compared to women with normal cytology. These patterns remained when the comparison was limited to the three DNA assays.

**Table 3:** Horizon samples: Positive agreement between Hybrid Capture 2, cobas, CLART, and APTIMA, by age, screening history, and concurrent cytology.

	Samples with allhuman papillomavirustests	Age^a^		Screening history (age 30 to 65 years)		Cytology (primary samples, age 30 to 65 years)^b^	
		23 to 29 years	30 to 65 years	Primary samples	Follow-up samples	Normal	Abnormal^c^
**Total**	5,064 (100%)	1,534 (100%)	3,256 (100%)	2,881 (100%)	375 (100%)	2,741 (100%)	127 (100%)
Hybrid Capture 2, cobas, CLART,and APTIMA: at least one positive	1,679 (33.2%)	744 (48.5%)	800 (24.6%)	655 (22.7%)	145 (38.7%)	562 (20.5%)	93 (73.2%)
Hybrid Capture 2, cobas, andCLART: at least one positive	1,636 (32.3%)	731 (47.7%)	771 (23.7%)	630 (21.9%)	141 (37.6%)	537 (19.6%)	93 (73.2%)
**Four human papillomavirus DNA or mRNA assays: Hybrid Capture 2, cobas, CLART, and APTIMA**							
At least one positive	1,679 (100%)	744 (100%)	800 (100%)	655 (100%)	145 (100%)	562 (100%)	93 (100%)
One positive	470 (28%)	136 (18%)	303 (38%)	261 (40%)	42 (29%)	248 (44%)	13 (14%)
Two positive	268 (16%)	125 (17%)	130 (16%)	104 (16%)	26 (18%)	100 (18%)	4 (4%)
Three positive	260 (15%)	117 (16%)	124 (16%)	102 (16%)	22 (15%)	89 (16%)	13 (14%)
All four positive	681 (41%)	366 (49%)	243 (30%)	188 (29%)	55 (38%)	125 (22%)	63 (68%)
**Three human papillomavirus DNA assays: Hybrid Capture 2, cobas, and CLART**							
At least one positive	1,636 (100%)	731 (100%)	771 (100%)	630 (100%)	141 (100%)	537 (100%)	93 (100%)
One positive	460 (28%)	134 (18%)	295 (38%)	252 (40%)	43 (30%)	239 (45%)	13 (14%)
Two positive	324 (20%)	150 (21%)	157 (20%)	130 (21%)	27 (19%)	118 (22%)	12 (13%)
All three positive	852 (52%)	447 (61%)	319 (41%)	248 (39%)	71 (50%)	180 (34%)	68 (73%)

^a^ Age groups targeted by the Danish cervical screening program.

^b^ Cytologically inadequate samples (n = 13) were not included.

^c^ Atypical squamous cells of undetermined significance or worse. Atypical squamous cells of undetermined significance (n = 63): 62% (n = 23) concordant on all four assays, and 70% (n = 26) on all three DNA assays. Low grade squamous intraepithelial lesions (n = 32): 56% (n = 15) concordant on all four assays, and 59% (n = 16) on all three DNA assays. High grade squamous intraepithelial lesions or worse, including atypical squamous cells cannot exclude high grade squamous intraepithelial lesions, atypical glandular cells, adenocarcinoma in situ, and carcinoma (n = 32): 86% (n = 25) concordant on all four assays, and 90% (n = 26) on all three DNA assays.

Virtually all kappa coefficients for pairwise agreement were ≥0.60, suggesting good overall agreement between the four assays (Table 4). Yet, only 52% of primary samples from women aged 30 to 65 years testing positive on either Hybrid Capture 2 or cobas were positive on both. When comparing Hybrid Capture 2 with CLART and APTIMA, these figures were 50% and 58%, respectively. In total, 8.7% of these primary samples were discordant on Hybrid Capture 2 and cobas, 9.2% on Hybrid Capture 2 and CLART, and 5.7% on Hybrid Capture 2 and APTIMA. Discordant samples between cobas and CLART constituted 8.5% of primary samples from women aged 30 to 65 years, 9.7% between cobas and APTIMA, and 10.3% between CLART and APTIMA. Cytology was abnormal in 4.4% of the same primary samples (Table 5).

**Table 4:** Horizon samples: Agreement between cobas, CLART, APTIMA, and Hybrid Capture 2 assays.

			Agreement (%)		Disagreement (%)				
Assay A		Assay B	Assays A and B negative	Assays A and B positive	Assay A positive, B negative	Assay A negative,B positive	Total	Positive agreement (95% CI)	Kappa coefficient (95% CI)
Column 1		Column 2	Column 3	Column 4	Column 5	Column 6	Column 7 [ = C5+C6]	Column 8 [ = C4/(C4+C7)]	Column 9
**All samples (n = 5,064)** a									
Hybrid Capture 2	Cobas		3,591 (70.9%)	921 (18.2%)	114 (2.3%)	435 (8.6%)	549 (10.8%)	62.7% (60.2–65.1)	0.70 (0.68–0.72)
Hybrid Capture 2	CLART		3,619 (71.5%)	874 (17.3%)	160 (3.2%)	399 (7.9%)	559 (11.0%)	61.0% (58.5–63.5)	0.68 (0.66–0.71)
Hybrid Capture 2	APTIMA		3,929 (77.6%)	747 (14.8%)	288 (5.7%)	99 (2.0%)	387 (7.6%)	65.9% (63.1–68.6)	0.75 (0.73–0.77)
Cobas	CLART		3,507 (69.3%)	1,085 (21.4%)	270 (5.3%)	188 (3.7%)	458 (9.0%)	70.3% (68.0–72.6)	0.76 (0.74–0.78)
Cobas	APTIMA		3,628 (71.6%)	769 (15.2%)	587 (11.6%)	77 (1.5%)	664 (13.1%)	53.7% (51.1–56.2)	0.62 (0.59–0.65)
CLART	APTIMA		3,672 (72.5%)	738 (14.6%)	535 (10.6%)	107 (2.1%)	642 (12.7%)	53.5% (50.8–56.1)	0.62 (0.59–0.64)
**Primary samples, 30 to 65 years (n = 2,881)** b									
Hybrid Capture 2	Cobas		2,353 (81.7%)	275 (9.5%)	62 (2.2%)	190 (6.6%)	252 (8.7%)	52.2% (47.9–56.4)	0.64 (0.59–0.68)
Hybrid Capture 2	CLART		2,343 (81.3%)	262 (9.1%)	74 (2.6%)	192 (6.7%)	266 (9.2%)	49.6% (45.4–53.9)	0.60 (0.56–0.65)
Hybrid Capture 2	APTIMA		2,494 (86.6%)	222 (7.7%)	115 (4.0%)	49 (1.7%)	164 (5.7%)	57.5% (52.6–62.4)	0.70 (0.65–0.74)
Cobas	CLART		2,290 (79.5%)	337 (11.7%)	127 (4.4%)	117 (4.1%)	244 (8.5%)	58.0% (54.0–62.0)	0.68 (0.64–0.71)
Cobas	APTIMA		2,372 (82.3%)	228 (7.9%)	237 (8.2%)	43 (1.5%)	280 (9.7%)	44.9% (40.6–49.2)	0.57 (0.53–0.61)
CLART	APTIMA		2,361 (82.0%)	214 (7.4%)	240 (8.3%)	56 (1.9%)	296 (10.3%)	42.0% (37.7–46.2)	0.53 (0.48–0.57)

Note: Samples returning an invalid outcome on one or both compared assays were excluded, hence row totals do not add up to 5,064 (for all samples) or 2,881 (for primary samples in women aged 30 to 65 years).

^a^ In total, 371 (7.3%) samples had abnormal cytology.

^b^ In total, 127 (4.4%) samples had abnormal cytology.

**Table 5:** Review of the literature: Agreement between cobas, CLART, APTIMA, and Hybrid Capture 2 assays.

							Agreement		Disagreement			
Authors, publication year	Assay	Age group (years)	Total sample number	% Atypical squamous cells of undetermined significance or worse	% Hybrid Capture 2 positive	% Assay positive	Both negative (%)	Both positive (%)	Hybrid Capture 2 negative/Assay positive (%)	Hybrid Capture 2 positive/Assay negative (%)	% Total disagreement	Positive agreement (95% CI)
Column 1	Column 2	Column 3	Column 4	Column 5	Column 6	Column 7	Column 8	Column 9	Column 10	Column 11	Column 12 [ = (C10+C11)/C4]	Column 13 [ = C9/(C9+C10+C11)]
**Primary screening**												
Horizon^a^	Cobas	30 to 65	2,881	4%	12%	16%	2,353 (81.7%)	275 (9.5%)	190 (6.6%)	62 (2.2%)	9%	52% (48–56)
Horizon^a^	CLART	30 to 65	2,881	4%	12%	16%	2,343 (81.3%)	262 (9.1%)	192 (6.7%)	74 (2.6%)	9%	50% (45–54)
Horizon^a^	APTIMA	30 to 65	2,881	4%	12%	9%	2,494 (86.6%)	222 (7.7%)	49 (1.7%)	115 (4.0%)	6%	58% (53–62)
Heideman et al., 2011^b^ [22]	Cobas	29 to 61	800	1%	6%	5%	745 (93.1%)	33 (4.1%)	10 (1.3%)	12 (1.5%)	3%	60% (47–73)
Cuzick et al., 2013 [23]	Cobas	20 to 66	6,000	5%	15%	16%	NR^c^	732^c^ (12.2%)	225 (3.8%)	174 (2.9%)	7%	65% (62–68)
Lloveras et al., 2013^b^ [24]	Cobas	23 to 89	898	NR	15%	14%	743 (82.7%)	101 (11.2%)	23 (2.6%)	31 (3.5%)	6%	65% (58–73)
Getman et al., 2009^d^ [27]	APTIMA	NR^d^	568	6%	11%	5%	505 (88.9%)	30 (5.3%)	1 (0.2%)	32 (5.6%)	6%	48% (35–60)
Wu et al., 2010 [21]	APTIMA	24 to 59	2,000	5%	17%	10%	1,646 (82.3%)	177 (8.9%)	24 (1.2%)	153 (7.7%)	9%	50% (45–55)
Cuzick et al., 2013 [23]	APTIMA	20 to 66	6,000	5%	15%	10%	NR^c^	553^c^ (9.2%)	68 (1.1%)	353 (5.9%)	7%	57% (54–60)
**Other settings (predominantly women referred for colposcopy after prior abnormalities)**												
Horizon^e^	Cobas	30 to 65	127	100%	68%	62%	35 (27.6%)	73 (57.5%)	6 (4.7%)	13 (10.2%)	15%	79% (71–88)
Horizon^e^	CLART	30 to 65	127	100%	68%	60%	36 (28.3%)	71 (55.9%)	5 (3.9%)	15 (11.9%)	16%	78% (70–87)
Horizon^e^	APTIMA	30 to 65	127	100%	68%	56%	40 (31.5%)	70 (55.1%)	1 (0.8%)	16 (12.6%)	13%	80% (72–89)
Stoler et al., 2011 [14]	Cobas	≥21	1,578	100%	32%	33%	990 (62.7%)	438 (27.8%)	76 (4.8%)	74 (4.7%)	10%	74% (71–78)
Gage et al., 2012 [5]	Cobas	≥30	1,824	34%	55%	58%	665 (36.5%)	893 (49.0%)	164 (9.0%)	112 (6.1%)	15%	76% (74–79)
Lindemann et al., 2012 [7]	Cobas	16 to 82	1,360	>80%	48%	46%	638 (46.9%)	561 (41.3%)	71 (5.2%)	90 (6.6%)	12%	78% (75–81)
Lapierre et al., 2012 [6]	Cobas	24 to 75	396	100%	33%	38%	233 (58.8%)	115 (29.0%)	34 (8.6%)	14 (3.5%)	12%	71% (64–78)
Wong et al., 2012 [12]	Cobas	15 to >60	466	34%	44%	42%	251 (53.9%)	186 (39.9%)	11 (2.4%)	18 (3.9%)	6%	87% (82–91)
Park et al., 2012 [8]	Cobas	NR	345	72%^f^	40%	33%	202 (58.6%)	108 (31.3%)	6 (1.7%)	29 (8.4%)	10%	76% (68–83)
Ki et al., 2013 [17]	Cobas	NR	146	58%	49%	37%	71 (48.6%)	50 (34.2%)	4 (2.7%)	21 (14.4%)	17%	67% (56–77)
Mesher et al., 2013 [18]	Cobas	18 to 67	∼668^g^	100%	80%	78%	NR	NR	NR	NR	NR	87%^g^ (NA)
Szarewski et al., 2008 [11]	CLART(hr)	26 to 36^h^	737	100%	NR	NR	NR	NR	58 (7.9%)	146 (19.8%)	28%	NR
García-Sierra et al., 2009 [13]	CLART(13)	15 to 80	367	76%	57%	41%	148 (40.3%)	143 (39.0%)	9 (2.5%)	67 (18.3%)	21%	65% (59–72)
Pista et al., 2011 [9]	CLART(13)	25 to 63	425	85%	64%	63%	151 (35.5%)	268 (63.1%)	0 (0%)	6 (1.4%)	1%	98% (96–100)
Szarewski et al., 2008 [11]	APTIMA	26 to 36^h^	949	100%	NR	NR	NR	NR	8 (0.8%)	109 (11.5%)	12%	NR
Reuschenbach et al., 2010 [10]	APTIMA	28 to 44^h^	275	>75%	69%	64%	75 (27.3%)	167 (60.7%)	10 (3.6%)	23 (8.4%)	12%	84% (78–89)
Clad et al., 2011 [4]	APTIMA	NR	424	64%	70%	65%	114 (26.9%)	261 (61.6%)	13 (3.1%)	36 (8.5%)	12%	84% (80–88)
Stoler et al., 2013 [16]	APTIMA	≥21	865	100%	49%	42%	433 (50.1%)	357 (41.3%)	10 (1.2%)	65 (7.5%)	9%	83% (79–86)
Mesher et al., 2013 [18]	APTIMA	18 to 67	∼1,200^i^	100%	80%	70%	NR	NR	NR	NR	NR	86%^i^ (NA)

The list of prior studies was compiled by searching for English-language papers in PubMed using keywords “cobas”, “CLART”/”Clinical Arrays”, or “APTIMA”, each in combination with “HPV”; the search was undertaken in June 2013. Studies were selected if cobas, CLART or APTIMA were compared to Hybrid Capture 2.

Abbreviations: hr = high risk not otherwise specified; NR = not reported; NA = not available.

^a^ Primary samples from women aged 30 to 65 years.

^b^ Women without cervical intraepithelial neoplasia grade two or worse.

^c^ Numbers of samples having an invalid outcome were not reported by pair of assays. Hence, numbers of samples testing positive on both assays were estimated from the reported totals of assay specific frequencies of positive outcomes, and the reported frequencies of discordant samples for pairs of assays; numbers testing negative on both assays were not calculated.

^d^ Non-denatured clinical specimens. Of the 568 samples, 56 were from women below age 30 years, and 512 from women aged ≥30 years who had human papillomavirus testing performed as part of routine screening in conjunction with a liquid based Pap.

^e^ Primary samples from women aged 30 to 65 years having atypical squamous cells of undetermined significance or worse on cytology (all recommended for colposcopy or for repeated testing).

^f^ Based on the total sample, n = 356.

^g^ Estimated based on the proportion of samples testing positive on cobas if Hybrid Capture 2 positive (90.6%; n tested in Predictors 2 study = 668), and the proportion of samples testing positive on Hybrid Capture 2 if cobas positive (95.1%; n tested in Predictors 1 and 2 studies = 1,199).

^h^ Interquartile range.

^i^ Estimated based on the proportion of samples testing positive on APTIMA if Hybrid Capture 2 positive (86.6%; n tested in Predictors 1 and 2 studies = 1,225), and the proportion of samples testing positive on Hybrid Capture 2 if APTIMA positive (98.6%; n tested in Predictors 1 and 2 studies = 1,199).

## Discussion

### Principal Findings

Horizon is among the largest studies to compare several human papillomavirus assays in primary screening. Although we found kappa coefficients suggesting a good level of agreement among pairs of the four commercially available human papillomavirus assays, our analysis of positive samples demonstrated substantial disagreement between the assays, particularly in primary screening samples from women aged 30 to 65 years. For all pairwise assay comparisons, there were roughly as many discordant as concordant positive samples. While 4% of these samples showed abnormal cytology, 6 to 10% were positive on one but negative on the other human papillomavirus assay.

Our analysis indicates that to fully elucidate the extent of disagreement between human papillomavirus assays, it is necessary to compare them on positive samples. The reason is that, even in a screening population with a high background risk of cervical cancer, a majority of samples test negative, and consequently discordant samples may have little impact on traditional measures such as the kappa coefficient. Similar limitations may apply to the relative sensitivity, relative specificity, and non-inferiority [19] of one assay against another. Our approach relies on the same principle as the calculation of the proportion of overall agreement, which is another commonly reported measure. Unfortunately, it has been rarely used on primary screening data [21], [22], [27], and in those studies attention has not been drawn to the implications of this type of analysis for the management of women with positive human papillomavirus tests.

While APTIMA detects E6/E7 mRNA from human papillomavirus infections, the other three assays detect viral DNA. Some disagreement in comparisons between the three DNA assays and APTIMA is therefore not surprising, yet the DNA assays showed more inter-assay disagreement than expected. Possible explanations for this finding include, firstly, that cobas and CLART, but also APTIMA, were run on a fixed volume input from the residual sample material. In contrast, Hybrid Capture 2 was run after re-suspension of the pelleted processed cytology material. Theoretically, the CE-IVD post-cytology processing protocol for Hybrid Capture 2 might have removed some free viral particles prior to human papillomavirus analysis and, consequently, the assay may have returned a lower proportion of samples with a positive human papillomavirus outcome. Whether the clinical performance of the assays was affected will be determined when histological outcomes become available. Secondly, the designs of the assays differ. While Hybrid Capture 2 relies on signal amplification from RNA probes to the entire human papillomavirus genome, cobas and CLART are DNA polymerase chain reaction amplification assays targeting L1 sequences of human papillomavirus genotypes. Thirdly, CLART (by our definition) and Hybrid Capture 2 were designed to detect 13 genotypes, and cobas to detect the same plus genotype 66. Samples positive only for genotype 66 though explain few discordant samples, with 11/190 (9%) of cobas positive/Hybrid Capture 2 negative, and 20/127 (16%) cobas positive/CLART negative primary samples from women aged 30 to 65 years showing infections with genotype 66 and none of the 13 high risk genotypes. Fourthly, assay cross reactivity to low risk genotypes, which could increase the positivity rate, might vary between the DNA assays. These cross reactivity profiles and their significance for discordant samples will be evaluated in a separate report. Fifthly, assay specific calibration of primers/probes for individual human papillomavirus genotypes might result in different analytical sensitivities for detection of infections. Consistent with this, the higher average human papillomavirus viral loads in younger women [28] and in women with dysplasia [29] might increase the positive agreement between assays, as indeed suggested by the patterns observed in our data (Table 3). A possible explanation is that human papillomavirus positive samples in young women or in women with cytological abnormalities have on average higher viral loads than the minimum detectable amount needed to return a positive result even by the comparably least analytically sensitive assays. By the same token, samples with a relatively low viral load might be those that are more susceptible to the set cut-off on any assay before these assays return a positive result. Therefore, a relatively high frequency of agreement between assays in young women and in women with abnormalities observed in our study could be a consequence of the fact that in these women samples with infections but a relatively low viral load are limited in number. In unselected screening samples, on the other hand, viral loads will be much more heterogeneous, representing everything from recent transient infections to high level persistent infections, allowing samples with lower viral loads having a more prominent role in determining the frequency of disagreement between the assays. Within the WHO proficiency testing panel, CLART was evaluated against known genome equivalents of genotype specific L1 plasmids. It detected the 13 high risk human papillomavirus genotype plasmids in copy number 50 to 500 genome equivalents per genotype [30]. For assays like Hybrid Capture 2 and cobas, a similar analysis would not be possible as they return a combined outcome for the targeted genotypes (cobas though also separately for genotypes 16 and 18). To increase the transparency of assay calibration for the targeted human papillomavirus genotypes, a call for international standards of calibration could be suggested.

### Strengths and Weaknesses of the Study

All samples were evaluated in the same laboratory by the same staff, trained and certified by the assays’ manufacturers, using testing protocols agreed upon prior to the study, and instrumentation and software as supplied by the manufacturers. Unlike in previous studies, samples were collected and stored in SurePath, and experts have called for an evaluation of new human papillomavirus assays using media other than PreservCyt [31].

Previously, 11,617 (primary) SurePath samples from the same area evaluated in the same laboratory were tested with Hybrid Capture 2 [32]. The median age of the women in that study was 36.4 years, and 6% had atypical squamous cells of undetermined significance or worse. The proportion of women aged 25 to 64 years testing positive on Hybrid Capture 2 was ∼17%, similar to the 16% in Horizon in the same subset of samples. Horizon results are therefore in good agreement with the earlier results from the same population.

Although 19% of the collected samples had to be discarded, a selection bias is unlikely as excluded samples were similar to included samples. There were no significant differences between the 5,064 included and the 1,194 excluded samples in terms of women’s age, cytology, and Hybrid Capture 2 outcomes, but follow-up samples were slightly more prevalent among the included than the excluded samples (Table 2).

Following the Danish routine recommendations, women with abnormal cytology were referred for colposcopy or for repeated testing. In addition, we are currently inviting women with positive human papillomavirus tests and normal cytology for repeated testing (about one year after the baseline test). False positive rates, clinical sensitivity, and clinical specificity of the assays will be reported upon completion of this histological follow-up. The lack of histological verification might appear as a weakness of the current report. However, this is not the case as screening programs have to implement follow-up procedures for all women with positive tests.

### Comparison with Previous Studies and Implications for Clinical Practice

Compared to studies from other geographical areas, the Copenhagen population has a relatively high prevalence of human papillomavirus infections. This can, however, not explain the reported inter-assay disagreement. This assertion is supported by the observation that samples with high prevalence rates of human papillomavirus infection, e.g. those with high grade abnormal cytology, showed better positive agreement than samples with lower prevalence rates. Furthermore, Horizon outcomes were similar to those from prior studies that reported concordant and discordant outcomes in populations with varying prevalence rates of human papillomavirus infection (Table 5). All prior studies used samples stored in media other than SurePath. For each study, we calculated the proportions of concordant and discordant samples, and the proportion of positive agreement (see Material and Methods).

Previous studies fell into two categories; a majority of studies based on samples from women followed up after prior cervical abnormalities, and a minority of studies based on primary screening samples. In all prior studies of women with abnormalities, about four out of five positive human papillomavirus samples were concordant between cobas, CLART or APTIMA, and Hybrid Capture 2, i.e. positive agreement was around 80%. Thus, like the in Horizon data for women with abnormal cytology, these prior studies showed relatively good agreement between the four human papillomavirus assays. In this respect, it seems to make little difference which human papillomavirus assay is chosen for use in triage. However, even in these women the assays did not always detect the same cervical intraepithelial neoplasia lesions. In one study, 101 (37%) of 273 cervical intraepithelial neoplasia grade two or worse lesions were missed by at least one of the seven compared assays, including Hybrid Capture 2, CLART, and APTIMA [11]. In a study including Hybrid Capture 2, cobas, APTIMA, and four other assays, 120 (33%) of 359 cervical intraepithelial neoplasia grade two or worse lesions were missed by at least one assay [33].

In studies of women attending primary screening, about one in two to two in three human papillomavirus positive samples were concordant between Hybrid Capture 2, and cobas, CLART or APTIMA (positive agreement range: 48 to 65%; Table 5). In these studies, the proportion of women with abnormal cytology varied from 1% to 6%, and the proportion of women testing positive on one human papillomavirus assay but negative on the other varied between 3% and 9%. The proportion of women with inter-assay disagreement was thus larger than the entire proportion of women with abnormal cytology. Both Horizon and prior studies thus clearly showed that for evaluation of human papillomavirus assay (dis)agreement, women with cervical abnormalities are not representative for those attending primary screening. The similarity of the results between the Horizon and prior studies suggests that the disagreement between the assays is neither population- nor storage media-specific, but might instead be related to variability in assay designs, and how the assays are calibrated to detect the targeted genotypes.

This analysis shows that although different human papillomavirus assays may return a roughly similar proportion of positive samples, on which basis it could be assumed that they also end up having a similar specificity for detecting lesions, they do not identify the same women as positive. In two ways these findings challenge the perception that human papillomavirus assays can substitute each other in primary screening. Firstly, cytology is a commonly recommended triage method in primary screening based on human papillomavirus testing. In our study, 127 out of the 2,881 primary screening samples from women aged 30 to 65 years were cytology abnormal defined as atypical squamous cells of undetermined significance or worse. Of these, 93 were positive on at least one human papillomavirus assay, but only 63 were positive on all four (Table 3). This means that if cytology triage was used as a basis for referral to colposcopy, 68% ( = 63/93) of the women would be referred independently of which of the four assays had been used for the primary screening. For the other women, 32% in our study, the type of follow-up and a possibility of referral would depend on the choice of the assay.

Secondly, by far the majority of the human papillomavirus-positive women are cytology normal. In this group, the disagreement between the four assays is even larger than in human papillomavirus-positive/cytology abnormal women. So, how should these women be managed? On the one hand, long-term follow-up studies have shown that human papillomavirus-positive/cytology normal women have a higher risk of high grade cervical intraepithelial lesions than human papillomavirus-negative women [1]. Hence, these women cannot be sent back to routine screening with the extended screening intervals proposed for human papillomavirus-negative women. On the other hand, a retesting interval of 6 or 12 months must be weighed against the burden of such an interim period for this large group of women, who, in absolute terms, still have a relatively low risk of high-grade cervical intraepithelial lesions, and for whom we know that they might likely have tested negative had they been screened with another human papillomavirus assay. The classic dilemma of how to manage human papillomavirus-positive/cytology normal women therefore becomes even more pertinent with the large degree of disagreement between different human papillomavirus assays when used in primary screening.

In conclusion, considerable concordance between the four human papillomavirus assays was observed for triage indications in women with abnormal cytology. However, in primary screening of women above age 30 substantial differences in detecting human papillomavirus infections were observed for the same assays. Knowing that the use of primary human papillomavirus testing could provide a number of benefits for cervical screening, this finding is nonetheless an unexpected challenge that will need to be addressed.
